# P184: Production and efficacy testing of antimicrobial fabrics for use in hospitals

**DOI:** 10.1186/2047-2994-2-S1-P184

**Published:** 2013-06-20

**Authors:** G Singh, JA Beddow, E Joyce, T Mason

**Affiliations:** 1 Sonochemistry Department (HLS), Coventry University, Coventry, UK

## Introduction

In recent years, hospital acquired infections (HAIs) have become a major issue of concern in the European health care system. The most common routes of transmission of HAIs are either by airborne routes or by direct contact. Direct contact can be mediated through many textile items including: bedding, clothing, wound dressings and curtains. Antimicrobial textiles could be used widely in health care environments in order to reduce the spread of HAIs.

## Objectives

The Sonochemistry centre at Coventry University is one of a group of organisations working on an EU FP7 funded project (SONO) to develop a new technology for producing antimicrobial textiles. This technology involves the use of an ultrasonic process (sonochemical) to coat fabrics with antimicrobial metal oxide nanoparticles (NPs). The aim is to produce such textiles for routine use in hospitals as bandages, hospital sheets and uniforms.

## Methods

The absorption method from ISO 20743:2007 was used for the determination of antibacterial efficacy using MRSA strain NCTC 10442 and *P. aeruginosa* strain NCTC 13359. Test samples consisted of polyester cotton coated with copper oxide (CuO) NPs. Sterile pieces of coated and uncoated fabric were inoculated with 10^5^ CFU/ml of bacteria and then incubated overnight at 37^o^C. The number of live bacteria on the samples post incubation was determined by plate counts.

## Results

Results indicated a good level of antibacterial activity against both MRSA and *P. aeruginosa*. The microbial population after 24 hours of incubation at 37°C attained 10^8^ CFU/ml on plain cotton controls while it did not exceed 10^5^ CFU/ml on the CuO NPs treated test fabric, indicating a 3 log reduction in microbial growth.

## Conclusion

The results have demonstrated that sonochemical synthesis and coating of CuO nanoparticles onto fabrics can be a useful method to produce antibacterial fabrics. In other work, not presented here, the testing has been extended to other metal oxide nanoparticles and other species of bacteria associated with nosocomial infections, with very positive results. This novel fabric may be useful for many applications in the healthcare and hygiene sector.

## Disclosure of interest

None declared

